# A cognitive analysis of deceptive pollination: associative mechanisms underlying pollinators’ choices in non-rewarding colour polymorphic scenarios

**DOI:** 10.1038/s41598-020-66356-4

**Published:** 2020-06-11

**Authors:** João Marcelo Robazzi Bignelli Valente Aguiar, Martin Giurfa, Marlies Sazima

**Affiliations:** 1 Programa de Pós-Graduação em Ecologia, Instituto de Biologia, Universidade Estadual de Campinas, 6109, Avenida Bertrand Russel, s/n, Cidade Universitária Zeferino Vaz - Barão Geraldo, Campinas, 13083-865 São Paulo Brazil; 2 Research Centre on Animal Cognition, Center for Integrative Biology, CNRS, University of Toulouse, F-31062 Toulouse, Cedex 09 France; 3 College of Animal Science (College of Bee Science), Fujian Agriculture and Forestry University, Fuzhou, 350002 China; 4 Institut Universitaire de France, Toulouse, France; 5 Departamento de Biologia Vegetal, Instituto de Biologia, Universidade Estadual de Campinas, 6109, Avenida Bertrand Russel, s/n, Cidade Universitária Zeferino Vaz - Barão Geraldo, Campinas, 13083-865 São Paulo Brazil

**Keywords:** Behavioural ecology, Decision

## Abstract

Intraspecific floral colour polymorphism is a common trait of food deceptive orchids, which lure pollinators with variable, attractive signals, without providing food resources. The variable signals are thought to hinder avoidance learning of deceptive flowers by pollinators. Here, we analysed the cognitive mechanisms underlying the choice of free-flying stingless bees *Scaptotrigona* aff. *depilis* trained to visit a patch of artificial flowers that displayed the colours of *Ionopsis utricularioides*, a food deceptive orchid. Bees were trained in the presence of a non-rewarding colour and later tested with that colour vs. alternative colours. We simulated a discrete-polymorphism scenario with two distinct non-rewarding test colours, and a continuous-polymorphism scenario with three non-rewarding test colours aligned along a chromatic continuum. Bees learned to avoid the non-rewarding colour experienced during training. They thus preferred the novel non-rewarding colour in the discrete-polymorphic situation, and generalized their avoidance to the adjacent colour of the continuum in the continuous-polymorphism situation, favouring thereby the most distant colour. Bees also visited less flowers and abandoned faster a non-rewarding monomorphic patch than a non-rewarding polymorphic patch. Our cognitive analyses thus reveal that variable deceptive orchids disrupt avoidance learning by pollinators and exploit their generalization abilities, which make them favour distinct morphs.

## Introduction

Many insects act as efficient pollinators because of their remarkable capacities to learn and memorize flower features associated with nectar and pollen reward^1^. These capacities underlie flower constancy, the ability to remain focused on a single flower species during foraging, as long as it remains profitable^2–4^. They play a fundamental role in the mutualistic relationship between insect pollinators and flowers as they ensure the collection of food and the fertilization of flowers. Yet what happens when one of the partners does not support a mutualistic interaction? A paradigmatic example of this situation is the so-called ‘deceptive pollination’, which occurs when flowers that do not provide food resources lure insect visitors by means of attractive sensory cues^5,6^. This phenomenon is common among orchids (Orchidaceae), in which a third of the species deceive their pollinators^7^ using diverse mechanisms^8^. The most common one is termed ‘generalized food deception’^8^. In this case, flowers present floral signals (e.g. visual, olfactory) that are attractive to a large number of floral visitors^6^. Generalized food deception is usually associated with intraspecific flower colour variation^5,6,9^, which is thought to be advantageous for deceptive plants as it may impede the learning of a specific colour as a predictor of the absence of resources by pollinators (‘Heinrich’s hypothesis’)^10^.

Colour variability in orchids can adopt different forms, such as a discrete polymorphism, with two or more clearly distinguishable morphs, or a continuous polymorphism, with high intraspecific variation and morphs distributed along a variation continuum^6^. Modelling of decision making by pollinators confronted with food-deceptive orchids suggested that the inability to discriminate between flowers morphs that are perceptually similar should favour discrete colour polymorphism^11^. In a continuous polymorphism scenario, pollinators would visit less deceptive flowers with intermediate colours as they would learn to avoid them faster based on their perceptual similarity. On the contrary, flowers with colours located at the extremes of the colour distribution would appear more distinct, and would be more visited, attaining thereby a higher reproductive success. On an evolutionary time scale, this would favour flowers with more discriminable colours and the emergence of discrete colour polymorphism^11^. These theoretical conclusions require experimental analyses to determine how pollinators perceive and make decisions in a food-deceptive scenario presenting either continuous or discrete polymorphic signals.

Achieving this goal is difficult as the only way to study decision making by foraging bees under laboratory conditions ensuring proper stimulus control, including the setting of artificial polymorphic colour scenarios, consists in luring the insects with sucrose solution. Yet, this is opposed to the food-deceptive scenario, which implies absence of reward. Moreover, depriving the insect visitors from reward would result in their ceasing of visits to the experimental site, and in the impossibility of addressing questions related to a deceptive scenario. Here we overcame this problem by training bees to forage on a rewarding neutral colour in the presence of non-rewarding colours, and testing them afterwards with known and novel non-rewarding colours. The test reproduces, therefore, the situation of a food-searching bee confronted with non-rewarding options of variable colour, and provides a valid approximation to a food-deception scenario.

Our experimental situation was inspired by the interaction between the neotropical deceptive orchid *Ionopsis utricularioides* (Sw.), which presents intraspecific floral colour variation^12^, and the stingless bee *Scaptotrigona* aff. *depilis* Moure 1942 (Meliponini), which is a floral visitor of this orchid^12^ and which can be trained to visit artificial feeders offering sucrose solution^13,14^. We reproduced colours available in *I. utricularioides* and trained bees to choose a rewarded neutral grey colour vs. non-rewarded orchid colours (henceforth CS+ and CS−, respectively), all presented on artificial flowers. This situation corresponds to a case of differential conditioning in which animals learn to discriminate stimuli with distinct outcomes^15,16^. We first asked if in addition to learning the excitatory properties of the rewarding grey colour (i.e. its attractive properties), bees also learn the inhibitory properties of the non-rewarding orchid colours (i.e. their deterring properties)^17,18^. In the first case, they should prefer the grey colour to a novel, neutral colour (CS_0_); in the second case, they should prefer the CS_0_ to a non-rewarding orchid colour. These decisions may be in turn dramatically affected by the perceptual similarity existing between the CS+, CS− and CS_0_.

We performed two experiments, in which the test situation following training corresponded to either a discrete colour polymorphism (two distinct colours available in the test) or a continuous colour polymorphism (three colours from a continuum). In a third experiment we contrasted these two polymorphic scenarios with a monomorphic one (i.e. all flowers displaying the same colour), comparing the number of visits and time required by a bee to quit a patch of the different scenarios after non-rewarding experiences. Our results reveal how bees driven by appetitive expectations, and having experienced negative outcomes on specific colours, react to novel unrewarding colours. They show that colour variability plays a key role in the strategy of deceptive orchids as it promotes further visits to colours different from the ones experienced as non-rewarding. Overall, we provide a cognitive laboratory analysis of the deceptive interaction between orchids and pollinators and uncover the perceptual and associative mechanisms underlying this ecological situation.

## Results

### Characterization and reproduction of *Ionopsis utricularioides* colours

**Figure 1:**
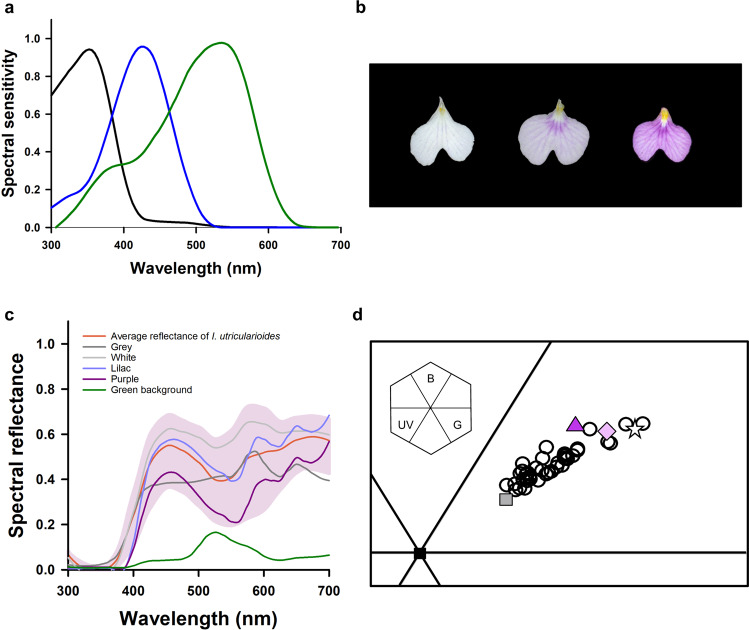
Colour analysis. (**a**) Spectral sensitivity curves of the three photoreceptor types of the stingless bee *Melipona quadrifasciata*. (**b**) Lips of *Ionopsis utricularioides* showing the continuous variation from white to purple for the human eye. (**c**) Spectral reflectance curves of the orchid flowers (the pink area represents the range between the max and min natural reflection curves; the red line represents the mean reflection), the artificial colours (grey, white, lilac and purple) and of the green background used in the experiments. (**d**) Loci of the natural and artificial colour stimuli in the colour hexagon, a generalized colour space for hymenopterans. Each open circle represents an individual orchid (n = 40). The other symbols correspond to the artificial colours (triangle: purple, diamond: lilac, star: white, square: grey). The inset shows the hexagon and its sections defined based on opponent processing of photoreceptor signals (B: blue; G: green; UV: ultraviolet).

We measured the spectral reflectance of the orchid flowers (see Methods for details) and calculated the loci of the colours measured in the colour hexagon, a generalized colour-opponent model proposed for hymenopterans^19^. As no electrophysiological measurements exist for the spectral photoreceptors of *Scaptotrigona* aff. *depilis*, we used those of another Meliponini as an appropriate choice in the light of the similarity found between the spectral sensitivity curves characterized for various bee species^20^ (Fig. 1a).

The flowers of *I. utricularioides* varied from white to purple to the human eye (Fig. 1b) and did not reflect in the range of ultraviolet (300–350 nm; Fig. 1c). They occupied the blue-green area of the colour hexagon (Fig. 1d). and presented a continuous floral colour polymorphism, where the more distant points in that space were separated by distances larger than 0.1 hexagon units (HU), which corresponds approximately to a discrimination level of 60% in bees^21^. Intermediate colour loci were separated from each other by distances smaller than 0.1 HU, thus suggesting a difficulty to discriminate them^22,23^. Flower colours varied between individual plants, but all flowers from the same plant presented the same colour.

Using the information obtained from the spectral reflectance measurements, we produced three colours similar to the ones displayed by the orchids using a colour printer (see Methods for details). The colours appeared white, lilac and purple to the human eye (Fig. 1c, Table 1). Furthermore, a grey colour was also printed to act as a “neutral stimulus” associated with food reward.

**Table 1:** Chromatic distances (in hexagon units) between the artificial colours used.

Stimulus	Grey	White	Lilac	Purple	Green Background
Grey	0	0.1032	0.1594	0.1921	0. 1627
White	0.1032	0	0.0508	0.1563	0. 4097
Lilac	0.1594	0.0508	0	0.0597	0. 2668
Purple	0.1921	0.1563	0.0597	0	0. 3275

Colour reflection was measured under the Plexiglas cover to quantify it as displayed for the bees in the different experiments. Chromatic contrast between each colour and the green background (last column) did neither influence learning nor test responses.

Figure 1c,d shows that the printed colours fell within the natural range of *I. utricularioide*s colours and were highly similar to them both in their spectral reflectance and in their loci in the hexagon space. Compared to the chromatic stimuli, the grey stimulus was closer to the centre of the hexagon, i.e. closer to the achromatic region of that space (Table 1). Figure 1c shows in addition the spectral reflectance curve of the green background on which the artificial flowers were presented.

### Colour choices in a non-rewarding, discrete polymorphic scenario

**Figure 2:**
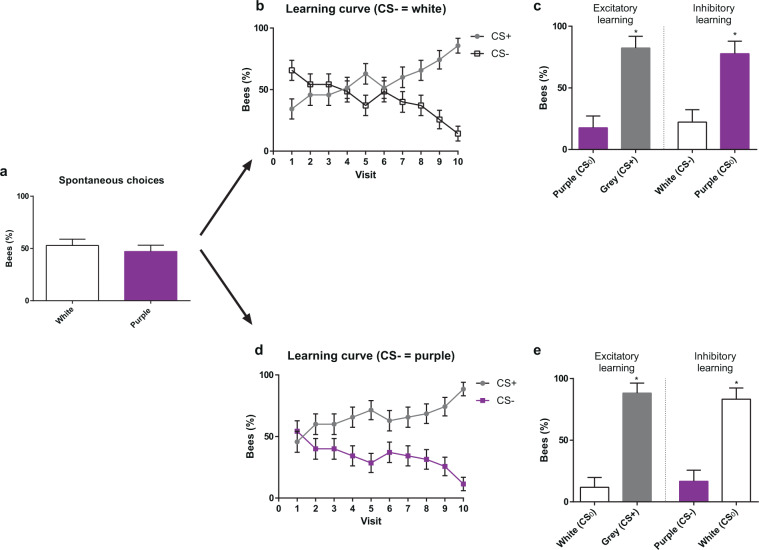
Colour choices in a non-rewarding, discrete polymorphic scenario. (**a**) Spontaneous choice of a white and a purple flower presented simultaneously. (**b**) Acquisition curves of bees trained along ten consecutive visits with three rewarded grey flowers (CS+) vs. three non-rewarded white flowers (CS−). (**c**) Tests opposing grey (CS+) to purple (CS_0_) (left) and white (CS−) to purple (CS_0_) (right). Bees preferred the CS+ over the CS_0_ and the CS_0_ over the CS−. (**d**) Acquisition curves of bees trained along ten consecutive visits with three rewarded grey flowers (CS+) vs. three non-rewarded purple flowers (CS−). (**e**) Tests opposing grey (CS+) to white (CS_0_) (left) and purple (CS−) to white (CS_0_) (right). Bees preferred the CS+ over the CS_0_, and the CS_0_ over the CS−. *: P < 0.05. Error bars represent the standard error of the mean (s.e.m.).

The experiment started with a spontaneous-preference test, in which each individually marked *Scaptotrigona* bee was presented with two non-rewarding artificial flowers, each displaying a different colour, white or purple. A single choice was recorded per bee. No significant preference was found between these two colours (Fig. 2a; white = 52.85%, purple = 47.14%, *χ*^2^ = 0.4566, df = 1, *P* = 0.4992; n = 70).

After this test, each bee was trained to forage on a patch of six artificial flowers, three of which displayed the grey colour (CS+) and were rewarded with 50% (w/w) sucrose solution, while the other three displayed either the white or the purple colour (CS−) and were non-rewarded. Two groups were trained: one in which the CS− was white (n = 35) and another in which it was purple (n = 35).The chromatic contrast between the CS+ and CS− colours was higher in the case of grey vs. purple (0.1921 HU) than in grey vs. white (0.1032 HU; see Table 1). Each rewarded flower provided 300 µl of sucrose solution, which suffices to fill the crop of a foraging bee; non-rewarded flowers provided the same volume of water.

Each bee was recorded during 10 consecutive flower choices. If a bee landed on a non-rewarded flower, white or purple, it moved to a next flower until finding the sucrose reward. If it landed on the grey flower, it found the sucrose solution and stayed there until filling its crop, returning afterwards to the hive. For each of the ten visits recorded, we computed the % of bees having chosen either the CS+ or the CS− flowers. Learning would be visible by a progressive increase of the % of bees visiting the grey flowers along the ten visits recorded.

Figure 2b,d shows the learning curves of the two groups of bees trained to choose the grey CS+ colour vs. their respective CS− colour, white or purple. Although the CS+ and the CS− curves mirror each other given the way in which performance was computed (a bee choosing a CS+ flower did not choose a CS− flower, see above), we presented both curves to provide a comparative view of how both groups of bees learned the discrimination. In both cases, the proportion of bees choosing correctly the grey rewarding flowers during the ten foraging bouts increased, decreasing thereby the choice of white / purple non-rewarding flowers (CS− white, *χ*^2^ = 31.252, df = 9, *P* < 0.01, n = 35; CS− purple, *χ*^2^ = 28.195, df = 9, *P* < 0.01, n = 35). In the last two visits, practically all the bees were going exclusively and directly to a grey flower, ignoring the CS− flowers. No significant differences were found in discrimination success between the two groups of bees (df = 9, *χ*^2^ = 8.1152, *P* = 0.522), which indicates that differences in chromatic contrast between the CS+ and the CS− colours did not influence discrimination learning.

After the tenth floral choice, all six flowers were removed and a test with two non-rewarded flowers was performed. Each group of bees was split in two subgroups to perform a single test per subgroup. During a test, a single choice was recorded per bee. In one test, one of the flowers presented the grey colour (CS+) and the other flower a novel colour (CS_0_), which was, in fact, the CS− of the alternative group of bees (i.e. purple for bees trained with grey vs. white, and white for bees trained with grey vs. purple). This test allowed to evaluate if, as expected, bees preferred the grey colour to the novel CS_0_ colour based on the excitatory properties of the CS+. The other test confronted the CS− colour (white or purple, depending on the experimental group) to the CS_0_ colour (purple or white, respectively). This test represents an approximation to a deceptive, discrete polymorphic scenario: the two colours were both non-rewarding and well distinguishable from each other. The test allowed evaluating if besides learning to choose the grey CS+ colour, bees also learned to avoid their CS− colour based on its inhibitory properties, and thus preferred to visit the novel CS_0_ colour.

In the test confronting the grey colour to the novel colour (Fig. 2c,e, ‘Excitatory learning’), bees preferred significantly the grey colour, which had been associated with food reward (CS− white: grey = 82.35%, purple = 17.64%; *χ*^2^ = 11.725, df = 1, *P* < 0.01, n = 17; CS− purple: grey = 88.23%, white = 11.76%; *χ*^2^ = 14.328, df = 1, *P* < 0.01, n = 17). This result shows that bees were guided by the positive expectations induced by the rewarding grey colour during training.

In the test confronting the CS− colour to the CS_0_ colour (Fig. 2c,e, ‘Inhibitory learning’), bees always preferred the novel colour to the non-rewarded colour (Figs. CS− white: white = 22.22%, purple = 77.77%, *χ*^2^ = 9.765, df = 1, *P* < 0.001, n = 18; CS− purple: purple = 16.66%, white = 83.33%, *χ*^2^ = 12.951, df = 1, *P* < 0.001, n = 18). This preference reveals that during the training, they learned the inhibitory properties of the CS−, which led them to avoid actively this stimulus and to choose the distinct novel stimuli during the test.

The latter result thus provides important insights to understand some aspects of a deceptive discrete polymorphic scenario. A foraging bee, driven by appetitive expectations, and having experienced a negative outcome at a coloured morph of a polymorphic orchid, will afterwards avoid that negative colour and prefer another morph displaying a distinct, discriminable colour. Colour variability thus further promotes visits to orchid flowers based on the learning of appetitive and aversive experiences during foraging.

### Colour choices in a non-rewarding, continuous polymorphic scenario

**Figure 3:**
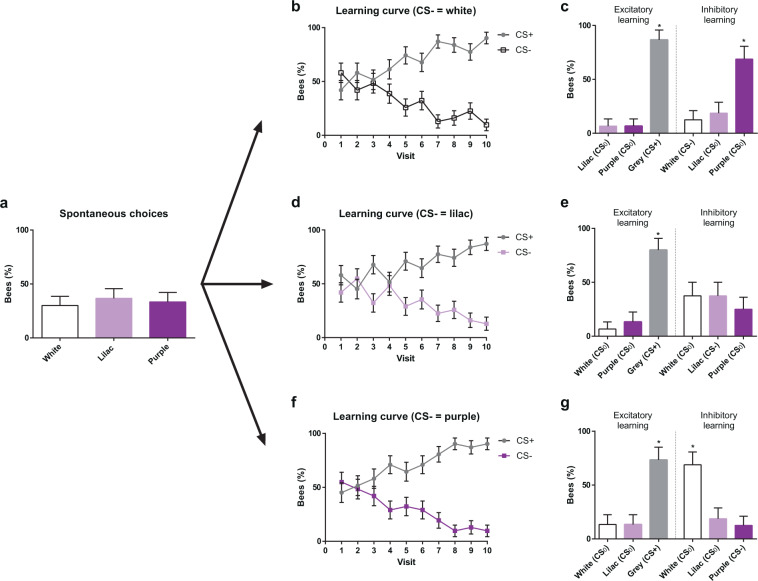
Colour choices in a non-rewarding, continuous polymorphic scenario. (**a**) Spontaneous choices of a white, a lilac and a purple flower presented simultaneously. (**b**) Acquisition curves of bees trained along ten consecutive visits with three rewarded grey flowers (CS+) vs. three non-rewarded white flowers (CS−). (**c**) Tests opposing grey (CS+) to lilac and purple (both CS_0_) (left), and white (CS−) to lilac and purple (both CS_0_) (right). Bees preferred the CS+ over the two CS_0_. When presented with the CS−, they preferred the CS_0_ (purple) that had the largest difference to the CS− white. In this case, the intermediate CS_0_ (lilac) was treated as the CS−. (**d**) Acquisition curves of bees trained along ten consecutive visits with three rewarded grey flowers (CS+) vs. three non-rewarded lilac flowers (CS−). (**e)** Tests opposing grey (CS+) to white and purple (both CS_0_) (left), and lilac (CS−) to white and purple (both CS_0_) (right). Bees preferred the CS+ over the two CS_0_. They distributed equally their choices between the CS− lilac and the two adjacent CS_0_ colours, thus showing inhibitory generalization from the CS− to the CS_0_ colours. (**f**) Acquisition curves of bees trained along ten consecutive visits with three rewarded grey flowers (CS+) vs. three non-rewarded purple flowers (CS−). (**g**) Tests opposing grey (CS+) to white and lilac (both CS_0_) (left), and purple (CS−) to white and lilac (both CS_0_) (right). Bees preferred the CS+ over the two CS_0_. When presented with the CS−, they preferred the CS_0_ (white) that had the largest difference to the CS− purple. The intermediate CS_0_ (lilac) was treated as the CS−. *P < 0.05. Error bars represent the standard error of the mean (s.e.m.).

The experiment started with a spontaneous-preference test, in which each bee was presented with three non-rewarding artificial flowers, each displaying a different colour, white, purple or lilac, and a single choice was recorded per bee. The three colours were aligned along a continuum in the colour hexagon, with lilac being intermediate between white and purple (see Fig. 1c). No significant preference was found for any of these three colours (Fig. 3a; white = 30.00%, lilac = 36.66%, purple = 33.33%, *χ*^2^ = 0.2993, df = 2, *P* = 0.861; n = 93).

After the spontaneous-preference test, three groups of bees were trained to forage on six artificial flowers, three of which displayed the grey colour (CS+) and were rewarded with 50% (w/w) sucrose solution, while the other three displayed either the white, the lilac or the purple colour (CS−) and were non-rewarded (n = 31 for each group).

Figure 3b,d,f shows the learning curves of the three groups of bees trained to choose the grey CS+ colour vs. their respective CS− colour, white, lilac or purple. As in the previous experiment, the % of bees choosing the grey rewarding flowers increased during the ten flower choices; accordingly, the choice of the CS− colour decreased proportionally (CS− white, *χ*^2^ = 30.088, df = 9, *P* < 0.01, n = 31; CS− lilac, *χ*^2^ = 21.801, df = 9, *P* < 0.01, n = 31; CS− purple, *χ*^2^ = 30.903, df = 9, *P* < 0.01, n = 31). No significant differences were found in discrimination success between the three groups of bees (*χ*^2^ = 11.6943, df = 18, *P* = 0.862), thus showing that differences in chromatic contrast between the CS+ and the CS− did not influence discrimination learning.

After finalizing the tenth flower visit, each group of bees was split in two subgroups to perform a single test per subgroup. In these tests, three non-rewarded flowers were presented and a single choice was recorded per bee. In one test, one of the flowers presented the grey CS+ colour and the other two flowers the novel CS_0_ colours (i.e. purple and lilac for bees trained with white as CS−, purple and white for bees trained with lilac as CS−, and white and lilac for bees trained with purple as CS−). In the other test, bees were confronted with their CS− colour vs. the two novel CS_0_ colours.

In the test confronting the grey colour to the two novel colours (Fig. 3c,e,g, ‘Excitatory learning’), bees preferred significantly the previously rewarded grey colour (CS− white: grey = 86.66%, lilac = 6.66%, purple = 6,66%; *χ*^2^ = 18.288, df = 2, *P* < 0.01, n = 15; CS− lilac: grey = 80.00%, white = 6.66%, purple = 13.33%; *χ*^2^ = 16.067, df = 2, *P* < 0.01, n = 15; CS− purple: grey = 73.33%, white = 13.33%, lilac = 13.33%; *χ*^2^ = 13.210, df = 2, *P* < 0.01, n = 15; see Supplementary Table S1). This result confirms that bees were guided by the appetitive outcome obtained on the grey colour during training.

In the test confronting the CS− colour to the CS_0_ colour (Fig. 3c,e,g, ‘Inhibitory learning’), bees exhibited significant preferences if their CS− was either white or purple, i.e. one of the extremes of the continuum (CS− white: white = 12.50%, lilac = 18.75%, purple = 68,75%; *χ*^2^ = 11.614, df = 2, *P* < 0.01, n = 16; CS− purple: white = 68.75%, lilac = 18.75%, purple = 12,50%; *χ*^2^ = 11.614, df = 2, *P* < 0.001, n = 16; Fig. 3c,g; see Supplementary Table S1). In these cases, the bees preferred the colour that was more distinct from the CS− (white for bees trained with purple as the CS− and purple for bees trained with white as the CS−; hexagon distances > 0.1 units). The intermediate colour lilac was treated as being similar to the CS− as responses to it did not differ significantly from those to the CS− (see Supplementary Table S1 for details). When the CS− was the intermediate colour lilac, the bees visited equally all three colours (CS− lilac: white = 37.50%, lilac = 37.50%, purple = 25,00%; *χ*^2^ = 0.740, df = 2, *P* = 0.690, n = 16; Fig. 3e), which indicates high generalization between these stimuli. These results reveal that during the training, the bees learned the inhibitory properties of the CS−, which led them to avoid this colour in favour of the more discriminable novel stimulus available during the test. Intermediate stimuli were assimilated to the CS− and treated similarly. When the CS− was the intermediate colour, inhibition was generalized to the two adjacent colours and the proportion of choices for the three alternatives was low.

The results of this experiment thus indicate again that a bee driven by appetitive expectations and having experienced a negative outcome in a non-rewarding coloured orchid morph, will avoid further flowers displaying the same colour, and will generalize this inhibition towards similar colours. On the contrary, it will orient its choice towards a morph distinctly coloured, favouring thereby colours at the extremes of the colour continuum.

### Comparing monomorphic and polymorphic colour scenarios: the number of visits and time spent exploring non-rewarding flowers

Colour variability may be an essential component to promote and maintain further visiting of a non-rewarded, variable floral species^10^. The degree of colour variability may directly translate into the time required by a pollinator to learn to avoid this species. To investigate this hypothesis, we compared the performance of bees foraging in a patch of artificial flowers displaying three distinct scenarios: i) a monomorphic situation, in which eight flowers presented the same colour (either white, purple or lilac), ii) a discrete polymorphic scenario with eight flowers, four of which were white and the other four purple, and iii) a continuous polymorphic scenario, in which nine flowers were presented to balance the spatial distribution of three colours (three purple flowers, three white flowers and three lilac flowers). Bees were previously pre-trained to visit the patch presenting eight rewarding artificial flowers during ten consecutive choices. During these pre-training visits, no colours were presented in the flowers, i.e. only the transparent Plexiglas flowers lay flat on the green background of the setup. After the tenth choice, and when the marked bee returned to the hive, the setup was cleaned and experimental conditions were changed to test its performance in one of the three scenarios described. For each scenario, we quantified the number of visited flowers and the total time spent (in seconds) at the experimental setup before abandoning it for more than one min. Bees usually required one min or less to return to the area from their nest.

**Figure 4:**
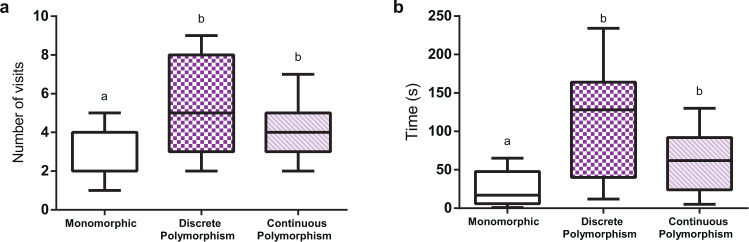
Number of visits and time before quitting a non-rewarding patch. Three scenarios are compared, all presenting non-rewarding flowers: i) a monomorphic patch with eight flowers presenting the same colour (either white, purple or lilac), ii) a discrete polymorphic patch with eight flowers, four of which were white and the other four purple, and iii) a continuous polymorphic patch, with nine flowers, three purple, three white and three lilac. (**a**) number of visited flowers and (**b**) time spent at the patch before quitting. Different letters above columns indicate significant differences. Error bars represent the standard error of the mean (s.e.m.).

Figure 4 shows the number of flowers visited and the time spent at the patch before abandoning it. For the monomorphic scenario, which could be either white (n = 15), purple (n = 15) or lilac (n = 15), no significant effect of stimulus identity was found (number of visits: *χ*^2^ = 0.1168, df = 2, *P* = 0.73; total time: *χ*^2^ = 0.0199, df = 2, *P* = 0.88), which allowed us to pool the data for this condition. In a monomorphic patch, bees visited relatively few flowers (2.56 ± 1.30; mean ± S.E.) before deciding to quit (Fig. 4a) and spent in average 26.13 ± 22.18 s while doing so (Fig. 4b). In both polymorphic scenarios (n = 15 each), on the contrary, bees visited more flowers (discrete polymorphism: 5.2 ± 2.3; continuous polymorphism: 4.26 ± 1.57) and spent more time at the patch before abandoning it (discrete polymorphism: 110.06 ± 72.08 s; continuous polymorphism: 62.80 ± 39.09 s). Comparing the three conditions thus revealed significant differences both for the number of flowers visited (Fig. 4a; *χ*^2^ = 20.376, df = 2, *P* < 0.001) and for the time spent at the patch (Fig. 4b; *χ*^2^ = 28.191, df = 2, *P* < 0.001). Differences were introduced by the monomorphic scenario as comparing the discrete and continuous polymorphic scenarios yielded no significant differences both for the number of visits and time spent at the patch (see Supplementary Table S2 for details).

Thus, after a rewarding experience at the patch (the pre-training period), the presence of colour variability in the artificial flowers promoted more visiting to non-rewarding flowers and increased the searching time at the patch. No differences between a continuous and a discrete polymorphic situation were found in our experimental situation.

## Discussion

We analysed the role of colour variability in deceptive pollination, using a cognitive approach, which focuses on perceptual and learning processes taking place in pollinators. Our study was directly inspired by the natural interaction between the deceptive orchid *Ionopsis utricularioides* and the stingless bee *Scaptotrigona* aff. *depilis*^12^. We reproduced floral colours and created polymorphic colour scenarios in an artificial patch of flowers to evaluate how stingless bees trained to collect food on a neutral grey colour react when they are confronted to non-rewarding flowers with known or unknown colours. As during training bees were presented with alternative, non-rewarding colours, our experimental design allowed determining if, and how, the impact of positive and negative experiences gathered during foraging translated into a test situation in which only non-rewarding flowers differing in colour were available. The latter scenario corresponds to that provided by the presence of deceptive polymorphic orchids differing in colour.

Our experiments showed that bees trained under a differential conditioning regime learn not only to choose the rewarded CS+ colour but also to avoid the CS− colour. They thus conform to the notion that in differential conditioning, animals learn both the excitatory and the inhibitory properties of stimuli with different outcomes^15,16^ Therefore, confronting the CS− colour vs. a novel CS_0_ colour resulted in a preference for the latter as a consequence of the avoidance induced by the former. In the discrete polymorphic colour scenario (Fig. 2), bees thus oriented their choices towards a novel test colour, whenever this colour was confronted with the colour previously experienced as non-rewarding. In an ecological context, the presence of two distinct colour morphs in discrete polymorphism will be of benefit for a deceptive orchid as a negative experience on one morph may induce further visitations to the alternative morph. In other words, deceptive pollination exploits in an efficient way, the cognitive capacities of pollinators.

Such exploitation is also revealed by the scenario of continuous colour polymorphism simulated in our work. In this case, we used three non-rewarding colours during the tests, one of which was the CS− previously experienced as non-rewarding. Colours were aligned in the colour space so that the two extremes of that line differed perceptually from each other but the intermediate colour was perceptually similar to the two extremes. In this colour constellation, another cognitive capacity, stimulus generalization, has to be considered. Generalization refers to an animal’s responding to stimuli that differ from a previously reinforced one but which are perceptually similar to it along a specific dimension (in this case chromatic distance in the colour hexagon)^24–27^. Upon differential conditioning, animals not only learn the excitatory and inhibitory properties of a CS+ and a CS−, respectively, but they also establish an excitatory and an inhibitory generalization gradient around them^17,28^. This means that stimulus attraction and avoidance can be transferred to novel stimuli based on perceptual similarity. In the discrete colour polymorphic scenario, generalization was reduced due to the chromatic dissimilarity of the two types of non-rewarding morphs. However, in the continuous polymorphic scenario, it played a significant role in the bees’ decisions when confronted to the three types of non-rewarding morphs. Inhibition was generalized from the extreme colours to the intermediate one, thus leading to a preference for the non-experienced extreme colour in the test situation. When the CS− was the intermediate colour, its inhibitory properties were generalized to both adjacent extremes in the colour continuum, thus reducing the number of visits to either morph. This result indicates that in a situation of continuous colour polymorphism, the extreme colours of a chromatic continuum will be favoured in detriment of the intermediate colours, which will be less visited based on inhibitory generalization.

This experimental result confirms previous theoretical assumptions from a mathematical model addressing the question of how the cognitive abilities of pollinators would influence decision making in a deceptive-pollination context^11^. The model predicted that under a continuous polymorphism, the association of a negative outcome with similar, intermediate colours would be facilitated due to the generalization capabilities of bees (see Fig. 3c,e,g). Our empirical approach addressed this hypothesis at the scale of an artificial patch of flowers and showed how intermediate coloured flowers would have a lower reproductive success, and flowers with more discriminable extreme colours would be favoured in the deceptive pollination context.

Our analysis of the temporal components of foraging in monomorphic vs. polymorphic (discrete and continuous) scenarios (number of visits and time spent before leaving the patch) revealed a significant effect in the case of colour monomorphism vs. colour polymorphism but not between discrete and continuous colour polymorphism. When only one non-rewarding colour morph was available, bees abandoned the patch earlier and performed less visits to non-rewarding flowers. When more than one type of non-rewarding flowers was presented, they took more time and inspected more flowers, thus confirming the advantage provided by colour polymorphism to deceptive orchids in terms of insect visitation probabilities. In theory, the scenario of discrete colour polymorphism should promote more visits than that of continuous colour polymorphism, simply because the former induces less generalization between non-rewarding morphs^11^. We were not able to detect such a difference, perhaps because the number of artificial flowers offered in our experimental situation (eight in discrete polymorphism and nine in continuous polymorphism) was too reduced to detect significant differences in the temporal parameters evaluated. Increasing the number of flowers in either case could reveal differences in these parameters between the two polymorphic scenarios.

Heinrich’s hypothesis suggested that the presence of intraspecific variation in floral traits of deceptive plants would favour deceptive pollination by impairing avoidance learning of non-rewarding food sources by pollinators^10^. Although we verified this in the case of colour information, the hypothesis may also apply to other variable traits such as flower scent^29^ or the presence of nectar guides^30^. The role of floral polymorphism in deceptive pollination was investigated in studies that did not find robust evidences supporting Heinrich’s ideas^9^. A meta-analysis of these studies concluded that polymorphism is a non-adaptive mechanism of relaxing stabilizing selection in floral traits^9^. In other words, the absence of food resources for pollinators would disrupt floral constancy^2^, relaxing thereby selective pressures on floral signals and promoting floral polymorphism. Yet, the analyses performed in these studies focused mainly on the plants, and relied on the quantification of variables such as fructification rates, which may be determined by factors such as the availability of nutrients and water, independently of any pollinator contribution^31,32^. Heinrich’s hypothesis includes considerations on the plants’ reproductive success, which was tested in the majority of the studies, but in Heinrich’s perspective, fruit formation depends strictly on the choices made by pollinators. It is, therefore, mandatory to evaluate the deceptive scenario through a consideration and understanding of the pollinators’ cognitive capacities rather than focusing exclusively on plant features. Generalized food deception by orchids relies on the exploitation of sensory biases and the disturbance of avoidance learning abilities of pollinators. Thus, not including these cognitive traits in ecological and evolutionary analyses of deceptive pollination may yield incomplete perspectives and conclusions.

## Methods

### Characterization and reproduction of *I. utricularioides* colours

Individuals of *I. utricularioides* were collected in Serra Azul, Brazil. They were individually kept in ceramic vases under 50% shade and watered every day. We measured the spectral reflectance of the flower labellum, using a USB4000 spectrophotometer (OceanOptics, Dunedin, FL, USA) calibrated between 300 and 700 nm and coupled to a deuterium-halogen light source (DH 2000; OceanOptics, Ostfildern, Germany). Reflectance measurements were calibrated using barium sulphate as a white standard and a black chamber as the black standard. We performed three measurements per flower, always at the right lobe of the labellum, in three flowers per plant. The angle between the surface measured and the light beam was of 45°.

Spectral reflectance curves were fed into the colour hexagon model, a generalized colour-opponent model proposed for hymenoptera^19^. For the calculation of chromatic distances, we included the standard illuminant function D65, the reflectance from the green surface used as background in behavioural experiments, and the spectral sensitivity curves of the photoreceptor types of *Melipona quadrifasciata* Lepeletier 1836^33^ as a proxy for those of *Scaptotrigona* photoreceptors^20^.

Colour loci and distances in the hexagon were calculated using the *pavo* package^34^ of R 3.4.2 (http://R-project.org). A threshold value of 0.1 hexagon units (HU) has been reported for colour discrimination^22,23^. We calculated the average distance value for each individual orchid and compared between individuals to evaluate floral colour polymorphism.

The function *spec2rgb* from the *pavo* package^34^ from R software was used to print colours similar to the ones displayed by the orchids. Colours were printed using a EPSON L3150 printer, on photographic paper (Smooth Pearl Paper, Ilford, Knutsford, UK), and their spectral reflectances were measured and represented in the colour hexagon, where their distances to the actual flower colours were estimated. All stimuli were measured after placing on top of them the Plexiglas used to build our artificial flowers, to quantify their reflection as displayed in our experiments. This did not affect our spectral measurements because our colour papers - like the real orchid flowers - did not reflect in the UV range, which is normally cut off by Plexiglas material.

The printed grey stimulus used as CS+ was discriminable from the other stimuli according to the hexagon model. The other printed colours were named according to the human vision: white, lilac and purple. While white and purple were discriminable from each other, lilac had an intermediate locus between these colours and was neither discriminable from white nor from purple (Fig. 1c, Table 1). All stimuli could be well discriminated from the green background (Table 1).

### Visual conditioning

*Scaptotrigona* aff. *depilis* individuals from four colonies present at the meliponary of the University of São Paulo, Ribeirão Preto, Brazil, were trained to visit a gravity feeder containing 50% (w/w) sucrose solution. The feeder was gradually moved away from the nest entrance to the experimental setup located 30 m away from the nest. The experimental setup was a patch of artificial horizontal flowers made of transparent Plexiglas and disposed on a green background. The flowers had a square base (3 cm × 3 cm × 0.5 cm) with a central cavity. A paper stimulus was placed on each base, and was covered by a Plexiglas square sheet. Both were perforated in the centre to allow access to the base cavity, which was filled with 300 µl of 50% (w/w) sucrose solution (rewarded flower) or water (non-rewarded flower). This volume allows a foraging bee to fill its crop in the case of a rewarding flower so that a single visit to a rewarding flower ended the foraging bout. On the contrary, visiting a non-rewarded flower resulted in more visits per foraging bout. Initially, the bees were allowed to perform ten bouts to all flowers with reward but without colour stimuli. At this time, bees were individually marked on the thorax, using a toothpick with the tip soaked in acrylic paint. We marked six bees at a time but kept only one for the experiment. The other five were removed from the setup by means of an aspirator (a plastic tube with a net in one of the ends) and enclosed within a plastic cage (16 × 13 × 9 cm). These bees were sequentially released after the previous bee completed the experimental sequence. They often came back to the setup searching for food, which allowed starting a new experiment with one of them. Unmarked bees appearing at the setup during the experiments were enclosed in a different cage. These bees were also released at the end of testing and could be later marked for further use. During a test for spontaneous preference preceding conditioning, two or three coloured flowers offering water were presented to the bees. When three flowers were presented, they were disposed in a row. During conditioning, more coloured flowers (numbers varied depending on the experiment) were available, some of them offering sucrose solution and some offering water. During tests, all flowers presented only water. The Plexiglas sheet was replaced after each bee visit, to avoid attractive or repellent scent marking^35^. After each visit to the arena, either for the tests before and after conditioning or for the training, the artificial flowers were randomly rearranged to avoid spatial learning. A visit was defined as landing on the flower surface followed by an insertion of the bee head into the central cavity of the flower.

### Experiment 1: Colour choices in a non-rewarding, discrete polymorphic scenario

Bees were first tested for their spontaneous preference between two unrewarded flowers, one white and the other purple. After this, they were divided into two groups. Both groups were trained in a differential conditioning procedure, with three flowers displaying a grey rewarded colour (CS+), and three non-rewarding flowers, either white or purple. Conditioning was performed with one bee at a time. Training finished when the bee completed ten rewarded visits.

After the tenth visit, all six flowers were removed and a test phase presenting only two non-rewarding flowers was performed. Trained bees were assigned to two subgroups to perform a single test per subgroup. The first test evaluated if bees preferred the grey colour to a novel CS_0_ colour based on excitatory learning of the CS+. The CS_0_ was the CS− experienced by the alternative group of bees (i.e. purple for bees trained with grey vs. white and white for bees trained with grey vs. purple). The second test evaluated if bees avoided the CS− colour and thus preferred to visit the CS_0_ based on inhibitory learning of the CS−.

Experiments were performed only during good-weather days. For a given experiment requiring two or more colour combinations, bees for each combination were trained during the same day to avoid influences of daily factors. For instance, for Experiment 1, which implied training a grey colour as the CS+ vs. either a purple or a white colour as the CS−, we alternated the training between these two combinations during the same day until completing the sample size of each group.

### Experiment 2: Colour choices in a non-rewarding, continuous polymorphic scenario

Spontaneous colour preferences were measured before training by presenting bees with three non-rewarded flowers, one white, one lilac and the other purple. Afterwards, three groups of bees were trained. All experienced three grey rewarded flowers (CS+), and three non-rewarded flowers, which could be either white, purple or lilac (CS−), depending on the trained group. After training, two non-rewarding tests presenting three flowers were performed. Each bee was tested in one of these tests. In one test, one flower presented the CS+ colour and the other two, two novel colours (CS_0_). In the other test, one flower presented the CS− colour and the other two, two novel colours (CS_0_). In both tests, CS_0_ colours were the CS− of the alternative groups trained.

### Experiment 3: Comparing monomorphic and polymorphic colour scenarios in terms of the number of visits and time spent exploring non-rewarding flowers

We trained individual bees to visit eight rewarding artificial flowers without colour and after the tenth visit, we replaced the sucrose solution by water in all eight flowers and added colours in order to create three different scenarios: a) monomorphism, in which all non-rewarding flowers presented the same colour (either white, purple or lilac), b) discrete polymorphism, in which four flowers were white and the other four were purple; and c) continuous polymorphism, in which nine flowers were presented, three purple, three white and three lilac. In each case, we quantified the number of visited flowers and the total time spent at the experimental setup (in seconds). For each bee, the experiment ended after the individual stopped visiting the patch for more than one minute. This period corresponds to the typical time spent by a bee between successive visits to the patch.

### Statistical analysis

Spontaneous choices were analysed using a χ^2^ test. During conditioning and tests of Experiment 1 and 2, flower visits were recorded as binomial events (0 for the absence of visit and 1 for the occurrence of visit). In the case of the conditioning phase, the proportion of bees that visited the CS+ or the CS− was analysed through a generalized linear mixed model (GLMM) for the binomial family, in which “Trial” was a continuous factor (trial effect) and “Individual Identity” (Bee) and “Date” (Replica) random effects (individual effect). In the case of Experiment 3, we analysed the number of visits and time spent visiting the patch using a GLMM for the Poisson family, in which “Individual Identity” (Bee) and “Date” (Replica) were random effects (individual effect). This analysis revealed that stimulus identity (i.e. colour) was not significant in the monomorphic scenario of Experiment 3. We thus pooled the data from the three groups trained, each with a different colour. Multiple comparisons were performed with the Tukey method (z values provided throughout). All statistical analyses were performed with R 3.4.2 software (http://www.R-project.org). We used the packages *lme4* and *lsmeans*^36,37^ for the GLMM and Tukey method, respectively.

## Supplementary information

Supplementary information .

## Data Availability

The datasets generated during and/or analysed during the current study are available in the Figshare repository, 10.6084/m9.figshare.12311135.
