# Supplementary material for: A cognitive analysis of deceptive pollination: associative mechanisms underlying pollinators’ choices in non-rewarding colour polymorphic scenarios
Source: Sci Rep. 2020 Jun 11;10:9476. doi: 10.1038/s41598-020-66356-4 (PMC7290031; doi:10.1038/s41598-020-66356-4)
Supplement: Supplementary file 1 — Supplementary information . [file 41598_2020_66356_MOESM1_ESM.pdf]

Supplementary Materials

A cognitive analysis of deceptive pollination: associative mechanisms underlying pollinators' choices in non-rewarding colour polymorphic scenarios

João Marcelo Robazzi Bignelli Valente Aguiar[1,2] *, Martin Giurfa[2,3,4,§] and Marlies Sazima[5,§]

[1] *Programa de Pós-Graduação em Ecologia, Instituto de Biologia, Universidade Estadual de Campinas, Campinas, São Paulo, Brasil*

[2] *Research Centre on Animal Cognition, Center for Integrative Biology, CNRS, University of Toulouse, F-31062 Toulouse Cedex 09, France*

[3] *College of Animal Science (College of Bee Science), Fujian Agriculture and Forestry University, Fuzhou 350002, China*

[4] *Institut Universitaire de France, France*

[5] *Departamento de Biologia Vegetal, Instituto de Biologia, Universidade Estadual de Campinas, Campinas, São Paulo, Brasil.*

*** Corresponding author: João Marcelo Robazzi Bignelli Valente Aguiar,** jmrobazzi@gmail.com

§ These authors share the senior authorship.

Table S1. Experiment 2 – continuous polymorphism: Statistical values of comparisons between colour responses for all groups (Tukey test). Significant comparisons are highlighted in red.

A) Excitatory learning (learning of grey as CS+)

Comparisons of test responses												
**Test comparisons**	Grey *vs.* White		Grey *vs.* Lilac		Grey *vs.* Purple		White *vs.* Lilac		White *vs.* Purple		Lilac *vs.* Purple	
	z	*P*	z	*P*	z	*P*	z	*P*	z	*P*	z	*P*
CS-: White	-	-	3.513	<0.01	3.513	<0.01	-	-	-	-	0	1
CS-: Lilac	-3.300	<0.01	-	-	3.269	<0.01	-	-	-0.598	0.821	-	-
CS-: Purple	-3.010	<0.01	3.010	<0.01	-	-	0	1	-	-	-	-

B) Inhibitory learning (learning of a CS- stimulus)

Comparisons of test responses						
**Test comparisons**	White *vs.* Lilac		White *vs.* Purple		Lilac *vs.* Purple	
	z	*P*	z	*P*	z	*P*
CS-: White	-0.484	0.878	-2.945	<0.01	-2.693	<0.05
CS-: Lilac	0	1	0.759	0.728	0.759	0.728
CS-: Purple	2.693	<0.05	2.945	<0.01	0.484	0.878

Table S2. Experiment 3: Statistical values of comparisons between variables (number of visits and time spent at the patch before quitting it) for all scenarios (Tukey test). M: monomorphic scenario; D: discrete polymorphism scenario; C: Continuous polymorphism scenario. Significant comparisons are highlighted in red.

Number of visits						Time					
M *vs.* D		M *vs.* C		D *vs.* C		M *vs.* D		M *vs.* C		D *vs.* C	
z	*P*	z	*P*	z	*P*	z	*P*	z	*P*	z	*P*
4.395	<0.01	3.005	<0.05	-1.173	0.469	4.996	<0.01	3.431	<0.05	-1.362	0.361
